# The use of digital escape rooms in nursing education

**DOI:** 10.1186/s12909-022-03879-6

**Published:** 2022-12-29

**Authors:** J. M. Rodríguez-Ferrer, A. Manzano-León, C. Fernández-Jiménez, J. M. Aguilar-Parra, A. J. Cangas, A. Luque de la Rosa

**Affiliations:** 1 Department of Psychology, Health Care Center, University of Almería, Almería, Spain; 2 Department of Psychology, University of Granada, Granada, Spain; 3 Department of Education, University of Almería, Almería, Spain

**Keywords:** Escape room, Gamification, Nursery education

## Abstract

Negative attitudes and prejudices towards people with a mental disorder are common across cultures and societies. The stigma associated with mental illness leads to a lower quality of life, given the discrimination and social exclusion suffered by people with this type of disorder. In the field of health, research has shown that doctors and nurses also manifest these types of stigmatizing behaviors and attitudes. The present study aims to create and apply an educational escape room for the purpose of training nursing students in mental health, promoting positive attitudes towards people who have a mental disorder. To do so, a pre-post study was conducted with an experimental group and a control group to determine whether the escape room was effective for the modification of stigmatizing behaviors compared to transmissive lecture class, and a third measurement was made at 6 months only to the experimental group to evaluate whether the changes produced by the escape room were maintained in the long term. The results indicate that the students participating in the study obtained better scores in sensitization and these remain better over time. It is concluded that the escape room used is suitable for the training and sensitization of future nursing professionals in the field of mental health, facilitating the learning of knowledge and positive attitudes towards severe mental disorder.

**Supplementary Information:**

The online version contains supplementary material available at 10.1186/s12909-022-03879-6.

## Introduction

In recent years, there has been a significant increase in mental disorders worldwide, a trend that according to different sources and institutions will increase as a result of COVID-19 [1]. Although there have been important advances in the care and approach to mental disorders, stigma and discrimination are still associated with these diseases, especially in the case of serious mental illnesses (SMI), which is common in all countries and cultures [2]. Stigma is a broad and complex concept that includes attitudinal and behavioral aspects. Corrigan et al. [3] consider that this construct comprises three components: first, the stereotypes or knowledge structures that allow us to categorize information about a social group; second, the prejudices or negative attitudes that are manifested towards the group; and third,, discrimination, which is the reaction or social consequence of prejudice [4, 5].

The manifestation of these negative consequences, such as exclusion or social marginalization, would be are produced the position of power of the group that stigmatizes against the group that is stigmatized [6]. Different studies have tried to analyze the characteristics of stigma in its cognitive, attitudinal, and behavioral dimensions. The results of these studies link general attributes such as aggressiveness, incompetence, poor self-control, or irresponsibility to the mental disorder, which generates attitudes of fear, rejection, or avoidance towards people who manifest this type of illness [7, 8].

The origin and maintenance of these attitudinal barriers are currently an important challenge for professionals dedicated to mental health, given their negative effects both on the person who manifests the disorder and on their family environment. These stigmatizing attitudes and behaviors lead to difficulties in interpersonal relationships, work, or studies, which would condition a lower level of well-being and quality of life [9, 10]..

Although stigma toward a person with a mental disorder is common and frequent in various fields and professions, the review of the literature on the subject highlights the interest dedicated to the manifestation of these discriminatory behaviors by professionals and students related to the field of health [11]. In this sense, research has analyzed whether these stigmatizing beliefs are more frequent and significant in these groups than in the general population [12]., as well as the consequences that these attitudes have on the care of the patient with a mental illness [11, 13], a situation that may even have an impact on diagnosis or even treatment [13, 14].

In the case of medical and nursing students, efforts have been made to design possible strategies to promote inclusive and non-discriminatory attitudes. Among these actions we find educational programs or courses that try to inform specifically about mental disorders [15], initiatives that are committed to promoting the change of attitudes through contact with individuals with mental disorders [11, 16, 17], or practices which combine both methods by deepening knowledge of these disorders through information and interaction with the collective [5, 18]. Studies conducted so far, however, conclude that the effectiveness of these actions has been limited. Recent research shows that a change in attitudes is possible through innovative training strategies such as virtual reality and video games [19, 20]. On the one hand, the study by Kolek et al. [19] conducted a pre-post-test investigation with a control group and an experimental group where they examined the effect of a narrative video game on implicit attitudes in young adults through the video game *Czechoslovakia 38-39: Borderlands*. Both groups had an intervention with video games, although the experimental group had a video game with a sensitizing load, while the control group had a strong narrative related to it but did not address the topic explicitly. Their results show long-term between-group attitude change in micro explicit attitudes but not macro explicit attitudes, suggesting that this may owe to the historical fact addressed. They consider that video games can tell personal stories about particular issues that humanize certain concepts and allow them to have a real impact on people, favoring attitudes of awareness about the topics addressed in the playful narrative. On the other hand, Bujíc et al. [20] conducted a between-subjects pre-test-post-test experimental laboratory study, distinguishing between a group that used virtual reality (VR), another using 2D videos, and another which used articles. The content was related to the refugee crisis of Syria through a narrative about the imaginary father of a refugee child. Their results show that immersive journalism (VR and 2D) has a greater effect on attitude towards human rights compared to written journalism, and, in the case of those students who felt more involved in the experience, it had a greater effect on positive attitudes towards human rights. These investigations reinforce the idea that the playful experience and the immersive narrative can be positive for changing attitudes towards social issues.

Although there is still little evidence on the effectiveness of these playful tools in changing attitudes towards SMI [21], the results of the studies carried out are very promising. In this sense, Cangas et al. [22] demonstrate the usefulness of a video game called Stigma-Stop to reduce stigma against people with mental disorders such as schizophrenia, depression, or bipolar disorder in high school students. The video game modified the negative attitudes and beliefs towards the mental illnesses of the students through the information offered about these disorders and the personal involvement of the student in the game. Mullor et al. [23] show that this same game, Stigma-Stop, reduces the stigmatizing attitudes and behaviors of Psychology students towards people with mental disorders.

Among the various types of video games, the escape room represents a highly valid tool to promote the change of attitudes because its dynamics have been proven to favor the learning of various skills, generating high motivation and interest in students [24]. Escape rooms consist of a track game in which players are locked in a room due to a narrative event and must solve a series of puzzles, unlock locks, and find hidden clues to escape at any given time, usually in less than an hour [25]. The active role of the student during this type of game favors the learning of information as well as various emotional competencies [19].

After an analysis of the recent literature, it has been observed that there is a very limited number of investigations that use playful strategies, such as escape rooms, for awareness of SMI which also observe their long-term effect. This study aims to expand the use of the escape room as a strategy to promote learning and awareness of mental illness and evaluates the impact of the escape room compared to a transmissive lecture class on nursing students, as well as the long-term effect of the escape room. The specific objectives of this research were:

1. To assess the escape room’s ability to reduce stigma for nursing students.
2. To determine whether escape rooms have an impact on academic performance and cooperative skills among students.
3. To assess whether the escape room has long-term effects on students and whether there are gender differences.

## Materials and methods

**Fig. 1:**
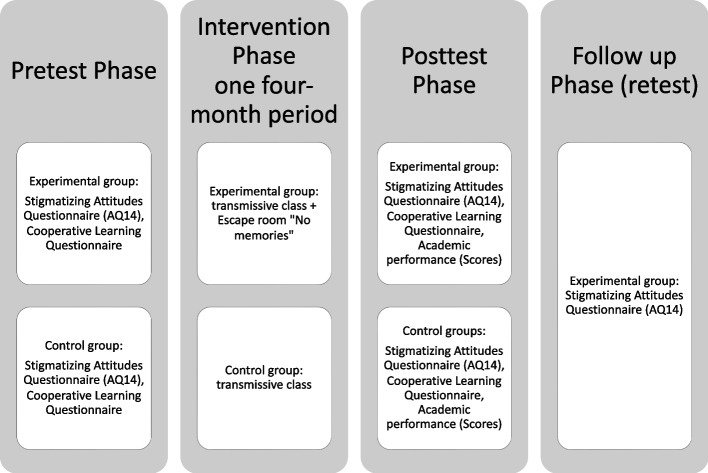
Experiment procedure

This research consists of a quasi-experimental longitudinal study, with a control and experimental group. The intervention was carried out in the subject of mental health in the nursing degree program. Both the control and experimental groups attended their regular classes, however, the experimental group participated in an escape room with sensitizing material on SMI. Fig. 1 shows a flowchart on the implementation of the research.

### Participants

The sample was selected using intentional non-probability sampling, as was convenience sampling, using the classes themselves as individual units. The sample initially included 6 classes from two universities in Andalusia (Spain).

The sample was made up of a total of 253 students with a mean age of 22.77 (SD = 6.85) years, of which 58 were men and 194 women. The experimental group had 138 participants, 35 men, and 103 women. Regarding the control group, it was made up of 115 people, 24 men, and 91 women. A majority of women is observed in the sample because the nursing sector in Spain is dominated by women [26].

Written and oral informed consent was gathered from the students. The methods used in this study were carried out in accordance with the Declaration of Helsinki. Ethics approval was obtained from the Research Ethics Committee of the University of Almería (UAL01/2021), Spain. Students were only excluded from the sample if they refused to give their informed consent to participate. The participants received neither economic nor academic incentives for taking part in the study.

### Instruments

#### Collaborative learning questionnaire [27]

This questionnaire measures the 5 dimensions of collaborative learning, social skills (α = .74), group processing (α = .75), positive interdependence (α = .72), promoter interaction (α =. 76) and individual responsibility (α = .79). In addition, it displays good psychometric properties not only in the factors as seen previously, but it also obtains an α = .89 as a whole. The model was validated by confirmatory factor analysis with satisfactory results S-B_χ_^2^ (165) = 3134.01, *p* < .001; * CFI = 0.942; * RMSEA (90% CI) = 0.040 (0.039-0.041); SRMR = 0.032.

2.2.2. Questionnaire on public stigma towards mental disorder AQ14 [28]. This questionnaire measures the stigma reported by people in 5 different factors: dangerousness, (ICC = .86), fear (ICC = .84), solidarity (ICC = .86), coercion (ICC = .85) and avoidance (ICC = .76). As can be seen, it shows good adjustment coefficients and adequate psychometry properties in the adjustment model according to confirmatory factor analysis S-B_χ_^2^ (193) = 1.326, *p* < .001; CFI = 0.983; RMSEA = 0.041 (0.039-0.041); SRMR = 0.042.

#### Academic performance

To evaluate this variable, the final exam score of a subject related to mental health was collected. The scores were established on a scale from 1 to 10, where the higher the score, the better the result.

### Escape room design

Escape rooms consist of a track game where players are locked in a room due to a narrative event and must solve a series of puzzles, unlock locks, and find hidden clues to escape at any given time, usually in less than an hour [25]. The active role of the student during this type of game favors the learning of information as well as various emotional competencies [19].

“No Memories” is an escape room where players must remember both who they are and where they are to escape from the room. Throughout the escape room challenges, the participants eventually discover that their character is a person with SMI, leading a normal life, displaying the characteristics and difficulties present in people with SMI.

Before starting the escape room, the students watched an explanatory tutorial video to understand how the platform works. The game design of the escape room follows a linear structure. Solving one clue will provide the object necessary to solve the next clue, and so on until the students escape. Tasks in a linear room must be solved in a particular order. The average duration of escape rooms is 60 minutes [29], so this educational playful experience was designed according to that duration. Additional file 1 shows in detail each quest and its relationship to awareness of a given serious mental illness.

Due to COVID-19, the intervention was carried out online and synchronously through the university’s learning platform. The students had to solve the escape room in teams of 4 people. Each of these groups was in a separate chat where the teacher and the researchers could enter to answer questions or provide clues. The students were offered all the clues they requested. This decision was made because all students had to finish the escape room in order to have all the information about the content taught.

### Procedure

After giving informed consent, the participants completed the questionnaires in an online classroom at the start of the course, while the researchers remained with the participants to administer the survey and to answer any questions. The researchers pointed out that all answers were anonymous and completely confidential. The students completed the questionnaires in a period of 20 minutes on average.

Next, the “No Memories” escape room was applied to the experimental group. Once each team had concluded, the participants were again given the questionnaires.

The participants in the control group attended and completed the subject on “Mental Health” in a traditional way (lectures and complementary work). They completed the questionnaires both at the beginning of the course and during the same period that the escape room was applied in the experimental group.

Finally, the experimental group answered the questionnaires again 6 months after the intervention.

### Data analysis

The data analysis was guided by the proposed research objectives. First, the factors of the instruments used was calculated. A Student’s t-test for independent samples was continued in the previous tests to determine whether the groups (control and experimental) were statistically equivalent. This was followed with another Student’s t for samples not related to the post-test balances with the intention of verifying whether there were statistical differences just after applying the programs. To determine what happened in the experimental group longitudinally, two Student’s t-tests were carried out for related samples, comparing the pre-test composition with the monitoring pressures and the pre-test pressures with the post-test pressures. To determine whether the program affects sensitivity towards stigmatizing attitudes differently according to gender, a MANOVA was carried out with the total AQ14 score in the follow-up proportions and the total proportions of the AQ14 pretest were included as covariates.

Finally, to measure the impact on academic performance, a Student’s t-test was conducted for independent samples with the score obtained in the subject in which the research was carried out.

## Results

To facilitate the reading of the results, all the means and standard deviations of the investigation are reported in Table 1. The pre-test, post-test, and follow-up (Retest) scores of the experimental group can be observed, as well as the pre-test and post-test scores for the control group. A necessary clarification for the “Stigmatizing Attitudes Questionnaire AQ14” is that a lower score in the questionnaire indicates lower stigmatizing attitudes and a higher score is a sign of more public discrimination towards people with SMI.

**Table 1:** Means and standard deviations of the variables studied in the control and experimental

	experimental group			control group	
	Pre-test	Post-test	Retest	Pre-test	Post-test
	M(SD)	M(SD)	M(SD)	M(SD)	M(SD)
Cooperative Learning Questionnaire					
Social skills	12.13 (3.11)	12.9 (3.27)		11.67 (2.69)	11.9 (2.51)
Group processing	11.58 (2.74)	12.48 (2.63)		12.23 (2.57)	12.16 (2.37)
Positive interdependence	11.77 (2.90)	12.72 (2.87)		11.97 (2.60)	11.55 (2.31)
Promoter interaction	11.79 (2.84)	12.85 (2.59)		11.78 (2.96)	11.27 (2.39)
Individual responsibility	11.96 (2.92)	12.82 (2.66)		11.89 (2.84)	11.43 (2.06)
Stigmatizing Attitudes Questionnaire AQ14					
Dangerousness	17.44 (6.07)	12.86 (4.39)	13.63 (4.86)	17.72 (5.13)	18.01 (4.97)
Solidarity	11.22 (4.07)	8.57 (2.43)	8.9 (2.65)	11.81 (2.39)	11.95 (2.41)
Coercion	8.13 (4.47)	6.29 (2.71)	6.52 (3.03)	9.12 (4.05)	9.65 (3.74)
Avoidance	11.36 (4.11)	8.06 (2.74)	8.57 (2.97)	12.06 (3.55)	12.23 (3.39)
Total Battery	48.15 (14.36)	35.78 (9.4)	37.61 (10.17)	50.71 (10.72)	51.83 (10.54)

Guided by the objectives of the investigation, the first step was to determine whether the groups (experimental and control) were equivalent. For this purpose, a Student’s t-test was carried out for independent samples with the pre-test scores of both questionnaires. These results were reported in Table 2 (in the pre-test columns), in addition to the results of the test statistic (column t) and the probability associated with it (column p). The effect sizes were reported using Cohen’s d (column d). The results of this test reveal that no statistically significant differences were found, thus it can be concluded that these groups are equivalent in the measured variables at the start of the intervention.

**Table 2:** Student’s t-tests for unpaired samples for control and experimental group

	Pre-test			Post-test		
	t	p	d	t	p	d
Cooperative Learning Questionnaire						
Social skills	1.248	.213	0.159	2.709	.007	0.35
Group processing	−1.951	.052	−0.247	1.019	.309	0.13
Positive interdependence	−0.547	.585	−0.070	3.511	.001	0.45
Promoter interaction	0.016	.988	.0002	5.016	.000	0.64
Individual responsibility	0.210	.834	0.027	4.548	.000	0.58
Stigmatizing Attitudes Questionnaire AQ14						
Dangerousness	−0.380	.710	−0.048	−8.714	.000	−1.10
Solidarity	−1.428	.172	−0.177	− 11.049	.000	− 1.40
Coercion	−1.843	.069	−0.232	−8.254	.000	−1.03
Avoidance	−1.441	.157	−0.181	−10.804	.000	−1.35
Total Battery	−1.622	.115	−0.203	−12.775	.000	−1.61
Academic Performance				3654	.000	0.46

Continuing with the data analysis, another Student’s t-test was performed for independent samples with the post-test scores (those obtained immediately after treatment in the groups). These results were reported in Table 2 in the columns corresponding to the right portion (post-test). In the results of this test, statistically significant differences are observed in all the variables except in “Group processing”. Although in the “Cooperative Learning Questionnaire” statistical differences were found in favor of the experimental group, if the effect sizes are considered (calculated using Cohen’s d) there are no major changes in the variables. Small effect sizes (d < .50) can be observed for “Social skills” and “Positive interdependence” and medium (0.50 < d < 0.80) for the variables “Promoter interaction” and “Individual responsibility”.

However, for the “Stigmatizing Attitudes Questionnaire AQ14” the changes that occurred in the factors of this questionnaire are of a significant magnitude and are considered large effect sizes (d > .80), in this case there is a statistical difference in stigmatizing public attitudes in favor of the experimental group.

In Table 2 the variable “Academic Performance” was also included to verify whether there were differences between the control and experimental groups. A Student’s t-test was performed for independent samples, and the results indicate that there are statistically significant differences, although the size of the effect found is small-medium.

In order to know the evolution of the experimental group in terms of the factors of public stigmatization, a follow-up measure (Retest) was carried out, which was compared with the pre-test and post-test score of the experimental group. The results of these Student’s t-tests for paired samples are reported in Table 3. Statistically significant differences were found in the two comparisons (pre-test measures with retest measures and post-test measures with retest measures). Upon comparing the pre-test measurements with those of the retest, it can be observed that the statistical changes found last 6 months after the completion of the program, with effect sizes ranging from medium (d < .50) to large (d > .80). By observing the right-hand columns of Table 3 (comparison between post-test and retest measures), it can also be seen that there are statistically significant changes. Essentially, what happened following the 6 months between these different tests is that the people who participated in the experimental group scored higher on the questionnaire. Although it is true that there are statistically significant differences when observing the effect sizes, it can be seen that the changes are of a very small magnitude according to the interpretation that can be attributed to Cohen’s d.

**Table 3:** Student’s t-test for paired (experimental group) samples between pre-treatment score and retest and post-treatment score and retest

	Pre-test-Retest			Post-test-Retest		
	t	p	d	t	p	d
Dangerousness	15.276	.000	−1.76	−7.463	.000	0.16
Solidarity	10.418	.000	−0.79	−4.945	.000	0.12
Coercion	7.978	.000	−0.50	−3.186	.000	0.07
Avoidance	13.946	.000	−0.93	−6.257	.000	0.18
Total Battery	14.984	.000	−1.02	−7.984	.000	0.18

Finally, a MANOVA was conducted in order to determine whether gender is a variable to be considered when carrying out this type of program. With this aim, the total score of the post-test of the “Stigmatizing Attitudes Questionnaire AQ14” was utilized, along with the total score obtained in the pre-test of this same questionnaire. The results of the Pillai F Trace statistic (1.421, *p* = .000) indicate that there are no differences by sex, which leads to the conclusion that the program affects both sexes in the same way.

## Discussion

People with severe mental disorders suffer great discrimination due to false beliefs and prejudices towards this group, which causes their marginalization and social exclusion [30]. The presence of these prejudices and negative stereotypes has been confirmed among professionals belonging to the healthcare field as well as among medical and nursing students [31]. The training of these future health workers must contemplate the learning of skills and attitudes that help to provide adequate care to people who have a mental disorder, especially those with serious mental disorders.

The present study seeks to demonstrate the benefits of using an innovative learning strategy – a digital escape room – to sensitize and train nursing students about serious mental disorders. While there is currently little evidence on the actual effectiveness of these online awareness programs [21], the enormous possibilities that video games have to increase knowledge about a topic and at the same time learn various skills can be highlighted [24]. This research shows how an escape room helps students empathize with the protagonist of the story and understand more experientially and actively the difficulties faced by people with severe mental disorders. These results coincide with previous research which establishes that through the dynamics and narrative established in the design of the escape room, contact and knowledge of other people’s life experiences is facilitated. This allows participants to reconsider their beliefs and prejudices about SMI [32, 33].

The results confirm that the experimental group that utilized the escape room obtained a statistically significant reduction of their stigmatizing attitudes compared to the students in the control group. Previous studies with video games also displayed similar results, showing that the use of the escape room is a good alternative to promote positive attitudes towards people with mental disorders [22, 23]. Traditionally, the strategies used to change attitudes in students have been focused on education and contact, so that knowledge about these illnesses and interaction with the person with a mental disorder decreases stigmatizing ideas and behaviors [15, 16].

These actions, however, are not incompatible with the use of video games. Instead, their use could be combined. For this reason, it would be interesting to study the effects that such initiatives would have on the reduction of stigma. On the other hand, in addition to its possibility to deepen the knowledge of a specific subject and its ability to sensitize and modify attitudes, it has been proven that the escape room has a positive impact on the motivation and relationship between university students, reasons that would amply justify its use as a didactic technique in the classroom [24].

As a limitation of the study, it must be noted that a non-probabilistic sample was used, however, a pretest was carried out which shows that both groups are equivalent. Also worth mentioning is the small size of the sample, which is also limited to the same geographical area. Future work could continue this line of research, in this case, by expanding the sample of the study. Another aspect to consider is the importance of designing a comparative study which applies the escape room along with other methodologies also used for awareness, such as a forum cinema, or talks given by professionals and/or by people with mental disorders. In this case, it would be interesting to know which of these practices is more effective in sensitizing for the change of attitudes, also distinguishing the one whose results last longer over time. Also, it would be worthwhile to determine what kind of playful elements within the escape rooms have the greatest effect on reducing stigma as well as whether there are differences between virtual and face-to-face escape rooms. Finally, future research could study other variables that could be modified by the escape room, such as prosocial behaviors and engagement, with standardized instruments, which would give a greater understanding of the use of the escape room as a tool for teaching innovation to raise awareness.

## Conclusions

Prejudices and stigmatizing behaviors towards people with mental disorders cause them to have a worse quality of life and their social relationships to be diminished. Therefore, it is important to raise awareness among the general population and specifically health professionals who may have contact with this population. This study presents the design and implementation of an escape room for SMI awareness for nursing students. The results show that the group that performed the escape room obtained a lower score in stigmatizing public attitudes compared to the control group. Therefore, it is concluded that this type of playful interventions where narratives and characters with SMI are shown can favor nursing students to become sensitized and have greater understanding of the subject.

## Supplementary Information

**Additional file 1.** The escape room presented can be found at the following link: x (Last access date 09/05/2022). Throughout the escape room, various clues appear which allow players to pass the tests. After reading the tests, in the event that the team does not know how to solve it, they can ask the facilitator as many times as they need for assistance.

## Data Availability

The datasets used and/or analysed during the current study available from the corresponding author on reasonable request.
