# Supplementary material for: The use of digital escape rooms in nursing education
Source: BMC Med Educ. 2022 Dec 29;22:901. doi: 10.1186/s12909-022-03879-6 (PMC9798354; doi:10.1186/s12909-022-03879-6)
Supplement: Supplementary file 1 — Additional file 1. The escape room presented can be found at the following link: x (Last access date 09/05/2022). Throughout the escape room, various clues appear which allow players to pass the tests. After reading the tests, in the event that the team does not know how to solve it, they can ask the facilitator as many times as they need for assistance. [file 12909_2022_3879_MOESM1_ESM.docx]

Additional file 1

The escape room presented can be found at the following link: x (Last access date 09/05/2022). Throughout the escape room, various clues appear which allow players to pass the tests. After reading the tests, in the event that the team does not know how to solve it, they can ask the facilitator as many times as they need for assistance.

Table 4. Guide on the implementation of the escape room

| Screenshot | Room | Dynamics and mechanics | Awareness elements |
| --- | --- | --- | --- |
| 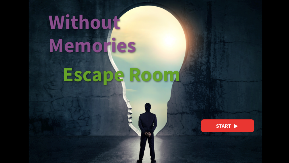 | Cover (Start of the escape room) | Escape room home page. Here the teams of 4 to 6 students will be formed, the rules will be explained, and they will have to press “Start” | None |
| 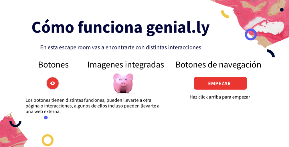 | Tutorial | The tutorial teaches how to use the Genially platform to properly enjoy the escape room |  |
| 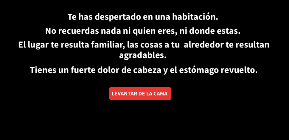 | Room 1 | Initial narrative message: “You have woken up in a room. You do not remember anything or who you are, or where you are. The place is familiar to you, the things around you are pleasant. You have a severe headache and an upset stomach.” They must hit the red button "Get out of bed" | The teams have just begun the narrative, they are expected to empathize with being a main character who does not know where he is. |
| 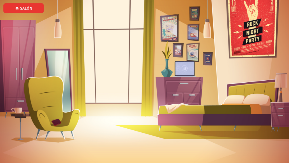 | Bedroom | Quest:   1. Find the protagonist's wallet where you can see his identification card with his name, Enrique Mañas León. 2. Arrange the arrows on the posters (which are enlarged if you hover over them with the mouse) on the wall according to when the radio, television, telephone, and alarm clock were invented. The solution is to unlock the computer in this room. | In the bedroom it is observed that the protagonist has ordinary hobbies (listens to rock, reads books, etc.) to eliminate the negative prejudice about normalized life within the SMI. |
| 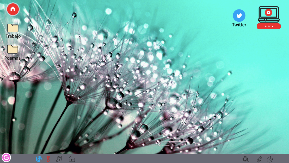 | Computer screen | Quest:   1. Caersar Cipher, is deciphered with the code found on a paper in the Hall | A short film on mental health is found on the computer (https://www.youtube.com/watch?v=cflepdPP-Sg) and the protagonist is shown again as an ordinary person with hobbies (poems) and work. <https://www.youtube.com/watch?v=cflepdPP-Sg> |
| 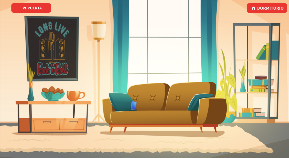 | Hall 1 | Quest:   1. Ceasar Code is deciphered with the code found on a computer. The solution is used to open the final safe. 2. Room 1 contains the protagonist's cell phone. To unlock it, it is necessary to read his diary, located in Room 2. | In the living room, it is again observed that the protagonist has ordinary hobbies (he listens to rock, reads books, etc.), which is done to eliminate the negative prejudice about normalized life within the SMI. |
| 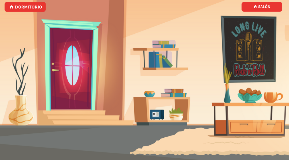 | Hall 2 | Quest:   1. Find Enrique's diary and decipher the hidden message in red letters to unlock the cell phone in Room 1. | The diary is one of the most important awareness-raising elements of the escape room. In it are testimonies of his daily life, adverse effects of medication, dealing with health personnel and the relationships he has with his friends and with a girl he likes. The diary is modifiable and any situation that you would like to reflect on with the university students could be presented. |
| 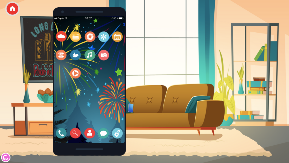 | Mobile phone | Quest:   1. After unlocking the phone, they have to observe that the name of one of the medicines that the protagonist takes appears. That, together with his age, will be the key to the Hall's safe. | On the mobile there is a second short film about SMI (https://www.youtube.com/watch?v=gK2Tmv4LfYI) and WhatsApp conversations with his nurse, his friend from a painting workshop and his crush (ordinary relationships are shown , as well as a cordial treatment from his nurse while they talk about erection problems due to medication).<https://www.youtube.com/watch?v=gK2Tmv4LfYI> |
| 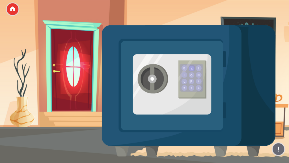 | Safe | Once the password is set, on this screen you just have to click on the house keys and go through the door. | |
| 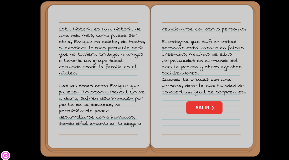 | Final message | When they go through the door, the teams have passed the escape room and a message appears with the following text: “Now you remember who you are. You had one too many drinks last night and it must have had a crossover effect with your medication. Today the 12th, you have a date with Cris, the girl you are meeting and luckily you will arrive on time. [NEXT ->] This story is a story of one more life, like any other. Enrique does not exist. In fact, if he existed, it would most likely be that he would not have a job, or friends, or would have a small social group where the family is the nucleus. People like Enrique who suffer from a Serious Mental Disorder, suffer daily discrimination from society, not allowing them to develop as individuals, making it difficult to find work or interact with other people. The stigma suffered by these people is based on false beliefs, many of them perpetuated in the world of cinema, the press, and other socializing agents. When you come across a person, give yourself the opportunity to meet them... you may be surprised. | |
